# Dyskerin and TERC expression may condition survival in lung cancer patients

**DOI:** 10.18632/oncotarget.4580

**Published:** 2015-07-16

**Authors:** Marianna Penzo, Vienna Ludovini, Davide Treré, Annamaria Siggillino, Jacopo Vannucci, Guido Bellezza, Lucio Crinò, Lorenzo Montanaro

**Affiliations:** ^1^ Department of Experimental, Diagnostic and Specialty Medicine, Alma Mater Studiorum, University of Bologna, Bologna, I-40138, Italy; ^2^ Department of Medical Oncology, S. Maria della Misericordia Hospital, Perugia, I-06156, Italy; ^3^ Department of Thoracic Surgery, University of Perugia, Perugia, I-06156, Italy; ^4^ Institute of Pathological Anatomy and Histology, University of Perugia, Perugia, I-06156, Italy

**Keywords:** lung cancer, dyskerin, TERC, TERC amplification, survival

## Abstract

Dyskerin mediates both the modification of uridine on ribosomal and small nuclear RNAs and the stabilization of the telomerase RNA component (TERC). In human tumors dyskerin expression was found to be associated with both rRNA modification and *TERC* levels. Moreover, dyskerin overexpression has been linked to unfavorable prognosis in a variety of tumor types, however an explanation for the latter association is not available. To clarify this point, we analyzed the connection between dyskerin expression, *TERC* levels and clinical outcome in two series of primary lung cancers, differing for the presence of *TERC* gene amplification, a genetic alteration inducing strong *TERC* overexpression. *TERC* levels were significantly higher in tumors bearing *TERC* gene amplification (*P* = 0.017). In addition, the well-established association between dyskerin expression and *TERC* levels was observed only in the series without *TERC* gene amplification (*P* = 0.003), while it was not present in *TERC* amplified tumors (*P* = 0.929). Similarly, the association between dyskerin expression and survival was found in cases not bearing *TERC* gene amplification (*P* = 0.009) and was not observed in *TERC* amplified tumors (*P* = 0.584). These results indicate that the influence of dyskerin expression on tumor clinical outcome is linked to its role on the maintenance of high levels of *TERC*.

## INTRODUCTION

Telomeres are tandem repeats of TTAGGG sequence, protecting the ends of chromosomes from deterioration or from fusion with other chromosomes. The length of telomeres is maintained only when sufficient levels of telomerase, the telomere replication enzyme, are expressed [1]. Telomerase is a ribonucleoproteic complex composed by the template sequence Telomerase RNA Component (TERC, also referred to as human telomerase RNA component, hTR), the enzyme telomerase reverse transcriptase (TERT) and a protein complex with protecting function on TERC, formed by dyskerin (the protein coded by *DKC1* gene), NOP10, NHP2 and GAR1. Most of the genes encoding telomerase components have been found to be mutated in different forms of Dyskeratosis Congenita (DC) (2), a rare multisystemic inherited syndrome, characterized by mucocutaneous abnormalities, predisposition to cancer and bone marrow failure, the latter being the principal cause of mortality (reviewed in [2–4]). Telomere shortening has been linked in many ways to carcinogenesis (reviewed in [5]), providing one of the possible explanations to the cancer predisposition typical of DC. On the other hand, in *DKC1* hypomorphic mouse, the only DC animal model available to date, an increase in breast and lung cancer occurrence has been described, which seems to be independent from telomere shortening, since it occurs when telomeres are still very long [6]. *DKC1* gene product, dyskerin, besides its role of TERC stabilization, is involved in ribosome biogenesis process; when its function is reduced, ribosomes show an altered translation of a subgroup of cellular mRNAs containing internal ribosomal entry site (IRES), whose de-regulation is well-described in cancer development [7]. The list of such genes includes those encoding the tumor suppressors p53 and p27 [8–9], the antiapoptotic factors Bcl-xL and XIAP [9] and the vascular endothelial growth factor (VEGF) [10].

Our group has found that dyskerin expression and functions are highly variable in human primary breast carcinomas in the general population: tumors characterized by low dyskerin expression also display reduced TERC levels and rRNA pseudouridylation, while the opposite is found in tumors expressing high dyskerin levels [11].

In a number of human tumor types of different origins, including breast, prostate, head and neck, colon and hepatocellular carcinomas [12] it has been reported that high levels of dyskerin expression are associated with an unfavorable prognosis. Since both ribosome biogenesis and telomerase function are known to be associated with disease specific survival [13–16], given the involvement of dyskerin in both of these processes in human tumors [11, 17], these findings are not surprising, indeed high dyskerin expression is likely to be associated with a very active ribosome biogenesis and high TERC levels requested for intense telomerase activity [17]. However, to date there is no evidence providing an explanation for the link between elevated dyskerin expression and poor prognosis. In the present study, we analyzed the connection between dyskerin expression, TERC expression and clinical outcome in two series of primary lung cancers, differing for the presence or absence of *TERC* gene amplification, a genetic alteration inducing strong TERC overexpression [18]. We found that *DKC1* expression influence on the clinical outcome is linked to its role on the maintenance of high levels of TERC.

## RESULTS

### Patients

The clinical and bio-pathological characteristics of the patients belonging to each series are reported in Table 1. At a mean follow-up time of 64.34 months (±6.92 SE), 40 patients (65.5%) had died: 33 (82.5%) deaths were due to disease recurrence and 7 (17.5%) to unrelated causes. Four (19.0%) of the 21 patients still on follow-up experienced recurrence: local recurrence was observed in 1 patient (25.0%), recurrence in lung and other sites in 3 patients (75.0%).

**Table 1:** Recapitulation of the clinical and bio-pathological characteristics of the two series of lung cancers collected

	TERC AMPLIFIED	TERC NON-AMPLIFIED
**TOTAL NUMBER**	30	30
**GENDER**		
Female	3 (10%)	4 (13.3%)
Male	27 (90%)	26 (86.7%)
**SMOKING STATUS**		
Non smoker	1 (3%)	4 (13%)
Smoker	29 (97%)	26 (87%)
Ex-smoker	0 (0%)	0 (0%)
**PATHOLOGICAL STAGE**		
IA	5 (17%)	6 (20%)
IB	11 (37%)	7 (23%)
IIA	0 (0%)	2 (6%)
IIB	5 (17%)	5 (17%)
IIIA	8 (26%)	5 (17%)
IIIB	1 (3%)	5 (17%)
**HISTOLOGICAL CLASSIFICATION**		
SCC	23 (77%)	13 (44%)
ADC	4 (13%)	9 (30%)
LCC	2(7%)	3 (10%)
BAC	0	1(3%)
Mixed	1(3%)	4 (13%)
**DYSKERIN EXPRESSION**	Range: 0.02–6.51 Median value: 0.21	Range: 0.03−2.13 Median value: 0.23
**MEAN OVERALL SURVIVAL**	62.60 (±9.69 SE)	63.88 (±9.36 SE)
**MEAN FOLLOW-UP TIME**	52.80 (±7.64 SE)	56.35 (±8.08 SE)
**MEDIAN AGE**	66.40 (±1.73 SE)	66.68 (±1.46 SE)

SCC: Squamous Cell Carcinoma; ADC: Adenocarcinoma; LCC: Large Cell Carcinoma; BAC: Bronchoalveolar Carcinoma.

### DKC1 and TERC expression are associated only in tumors not bearing TERC amplification

**Figure 1:**
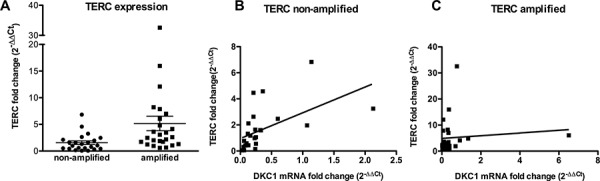
TERC and DKC1 expression in TERC gene –amplified or –non-amplified lung cancers **A.** Scatter plot graph showing TERC expression levels in the two series. TERC expression is significantly higher in *TERC* amplified tumors, as determined by Student's *t* test (*P* = 0.017) **B, C.** Correlation between TERC and DKC1 expression is direct in those tumors where *TERC* gene in not amplified (*P* = 0.003) (B) whereas there is no correlation in those tumors where *TERC* locus is amplified (*P* = 0.929) (C) as determined by linear regression analysis. TERC and DKC1 expression levels were measured by RT-PCR in cDNAs derived from two series of *TERC* gene –amplified and –non-amplified lung cancers, and compared to TERC expression in A549 cells.

Previous studies performed on tumors of different origin suggest that dyskerin expression reflects on the levels of pseudouridylation on rRNA and/or on telomerase function [11, 19–21]. On the other hand, TERT and TERC are frequently over-expressed in lung carcinomas [22], and there is evidence that *TERC* over-expression may be due to an amplification of 3q26, where *TERC* gene is mapped [23]. We analyzed a total of 60 lung cancers, 30 bearing an amplification of *TERC* gene and 30 bearing not, first of all by investigating how *TERC* locus amplification reflects on *TERC* expression levels. As expected, we found that *TERC* expression was significantly higher in those tumors where *TERC* locus was amplified (Figure 1A). Of note, no difference in dyskerin mRNA expression was observed comparing *TERC* amplified with *TERC* non-amplified group, indicating that TERC expression has no effect on dyskerin levels (Supplementary Figure S1). In addition, because of the known role of dyskerin on TERC stabilization, we wondered how TERC levels might be influenced by a conjunct effect of *TERC* locus amplification and *DKC1* expression in lung cancers. Our data showed that there was a significant direct correlation between DKC1 and TERC only in those tumors where *TERC* locus was not amplified, whereas the presence of *TERC* locus amplification, being associated to a strong TERC overexpression, completely abolished such a correlation (Figure 1B and 1C, respectively). Indeed, this correlation in *TERC* non-amplified tumors turned out to be present also when we considered smaller patient sub-groups, obtained by dividing the series in 2 stage-homogeneous groups (stage I and stages II–III). Conversely, in the *TERC*-amplified series, no correlation between DKC1 mRNA and TERC could be observed even comparing tumors of similar stages (Supplementary Figure S2). These data indicate that those tumors not bearing *TERC* gene amplification, and characterized by low dyskerin expression, also show low TERC levels, reasonably because the stabilizing activity of dyskerin on TERC leafs. On the other hand, in those tumors where *TERC* gene is amplified the abundance of TERC makes up for the lack of dyskerin stabilizing activity.

**Figure 2:**
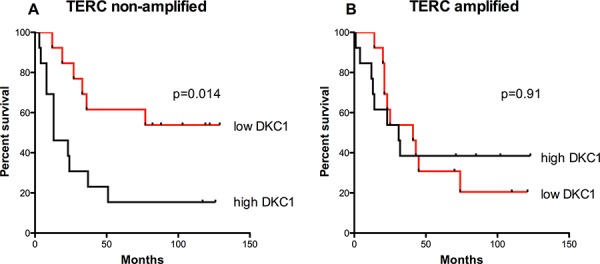
Overall survival is related to DKC1 expression only in those tumors where TERC gene is non-amplified **A, B.** Survival curves for *TERC* non-amplified (A) and *TERC*-amplified (B) lung cancer patients. DKC1 expression inversely correlates to overall survival only for those cases where *TERC* gene is non-amplified (*P* = 0.014) (A), whereas any correlation between DKC1 expression and overall survival is lost in those cases where *TERC* gene is amplified (*P* = 0.91) (B) The median value of DKC1 expression was chosen to divide cases between high and low dyskerin. Univariate analysis for overall survival was performed using the Kaplan and Meier approach, and the differences between curves were tested using the log-rank test.

### TERC amplification alters the association between dyskerin expression and survival

Previous studies by us and others have shown that high dyskerin expression negatively associates to prognosis in breast [11] and in hepatocellular [12] carcinomas: the higher dyskerin expression, the poorer the prognosis.

In line with these data, we found that in lung carcinomas the overall survival was significantly lower in patients with higher dyskerin expression compared to that of patients with lower dyskerin levels (Figure 2A). However, this observation was applicable only to *TERC* negative tumors; instead, in *TERC* amplified tumors, which are generally characterized by a poor prognosis, we found no significant correlation between dyskerin expression and overall survival (Figure 2B).

## DISCUSSION

In the present study, we found that, in lung carcinomas, the well-established association between dyskerin expression and TERC levels was observed only for those cases where *TERC* gene was not amplified. In accord, the association between dyskerin expression and survival was found only in those lung cancer cases not bearing *TERC* gene amplification.

The lack of correlation between dyskerin and TERC expression observed in tumors bearing *TERC* gene amplification can be well explained considering that in these cases the high transcription of *TERC* yielded to high amounts of TERC in the cells [18]. Reasonably, when the transcription rate is high enough, these effects could take place also independently of the stabilizing effect on TERC mediated by dyskerin.

High dyskerin expression in different tumor types resulted to be significantly associated with unfavorable prognosis [8, 12, 21, 24, 25]. On this regard, it has been proposed that this association could derive from the role that dyskerin plays in the maintenance of processes required for cancer cell growth, such as both telomere stabilization and ribosome biogenesis [7]. The present findings add an important piece of information to explain the role played by dyskerin overexpression in determining cancer cell behavior. Indeed, in lung cancer, the effect of dyskerin expression on prognosis can be ascribed mainly to its activity on TERC stabilization, since no significant repercussion on survival was observed in those tumors where no dyskerin/TERC relationship was found.

TERC is considered to be a non rate-limiting component of the telomerase complex and its amount is not generally quantitatively related to telomerase activity [26]. However, it may become a limiting component of the complex in those cases where TERT is highly overexpressed [27], thus conditioning the activity of the complex. Another possibility that should be taken into account is that TERC levels may play some role in those tumors where dyskerin is overexpressed independently of their telomerase activity, similarly to what is observed in CD4+ T cells, where it exerts a telomerase-independent anti-apoptotic function [28].

In conclusion the present study provides evidence that contributes to explain how those tumors overexpressing dyskerin, are characterized by increased aggressiveness and poorer prognosis.

## MATERIALS AND METHODS

### Patient material

We analyzed RNA extracted from two equally numerous series of consecutive patients selected from a previous study based on the presence or absence of *TERC* locus amplification [29].

These patients received a radical resection for primary Non Small Cells Lung Cancers at the Thoracic Surgery Unit of the Perugia University at S. Maria della Misericordia Hospital, Italy, between 2002 and 2006. Histological subtypes and grade of differentiation were determined according to the World Health Organization classification [30].

The only criteria used for patient selection was availability of tumor tissue from primary lung cancer and of survival data. Neither chemotherapy nor radiotherapy was administered before surgery. A follow-up, including a chest X-ray at 3 month intervals alternated with a total body Computed Tomography scan every six months, was scheduled for all patients for the first two years. Subsequently the patients underwent a Computed Tomography scan/year. Recurrences were detected by imaging techniques and when necessary confirmed by histological sampling. The use of patient material for this study was approved by the Institutional Ethics Committee and all patients gave their informed consent to participate in the study.

### Real time RT-PCR

RNA was isolated from frozen tumor tissue or by A549 cell line using the RNeasy Mini Kit on the QIAcube instrument (Qiagen s.r.l., Milan, Italy) according to the manufacturer's instructions. Then it was reverse-transcribed using the High Capacity cDNA Reverse Transcription kit (Applied Biosystems/Life Technologies Italia, Monza, Italy).

Real-time RT-PCR was performed as previously described [31]. Briefly, semi-quantitative Taqman approach (TaqMan Universal PCR master mix, Applied Biosystems) was employed to evaluate the expression of TERC, DKC1 and beta-glucoronidase as endogenous control. All real-time PCR reactions were carried out in triplicate in a Gene Amp 7000 Sequence Detection System (Applied Biosystems). Threshold cycles (C_t_) in each triplicate were averaged and fold differences compared to A549 expression levels were calculated by the ◮◮C_t_ method [32].

### Fluorescence *in situ* hybridization assay (FISH)

FISH assay was carried out on 4 μm (+1 μm) thick sections from formalin-fixed, paraffin-embedded tissue blocks from surgically resected tumor specimens of Non Small Cell Lung Cancer patients. The color TERC FISH probe was prepared with LSI TERC Spectrum Gold reagent (Abbott Molecular, Abbott Park, Illinois, U.S.A) according to the protocol previously described [33]. Analysis was performed on fluorescence microscope (Zeiss Axio Imager, Carl Zeiss S.p.A., Milan, Italy). For documentation, images were captured using a charge-coupled device camera (CoolSnap, Photometrics, Tucson, AR, USA) and merged using dedicated software (CytoVision, Leica Microsystems s.r.l., Milan, Italy). The scoring was carried out in 100 non-overlapping tumor cell nuclei per patient from four representative tumor areas. According to the Colorado criteria for epidermal growth factor receptor (EGFR) [33], the gene copy number for TERC was classified as increased (FISH-positive) when displaying gene amplification *[*>10% of tumor cells with >15 copies of the signals or gene clusters (>4 gene copies per cluster) or innumerable tight gene clusters] and high polysomy (≥40% of cells displaying ≥4 copies of the specific gene signal).

### Statistical analysis

Differences among groups were evaluated using the unpaired Student's *t* test. Correlations between continuous variables were computed by means of linear regression analysis. Univariate analysis for overall survival was performed using the Kaplan and Meier approach, and the differences between curves were tested using the log-rank test. *P* values below 0.05 were regarded as significant.

## SUPPLEMENTARY FIGURES


