# Supplementary material for: Dyskerin and TERC expression may condition survival in lung cancer patients
Source: Oncotarget. 2015 Jul 16;6(25):21755–60. doi: 10.18632/oncotarget.4580 (PMC4673301; doi:10.18632/oncotarget.4580)
Supplement: Supplementary file 1 [file oncotarget-06-21755-s001.pdf]

SUPPLEMENTARY FIGURES

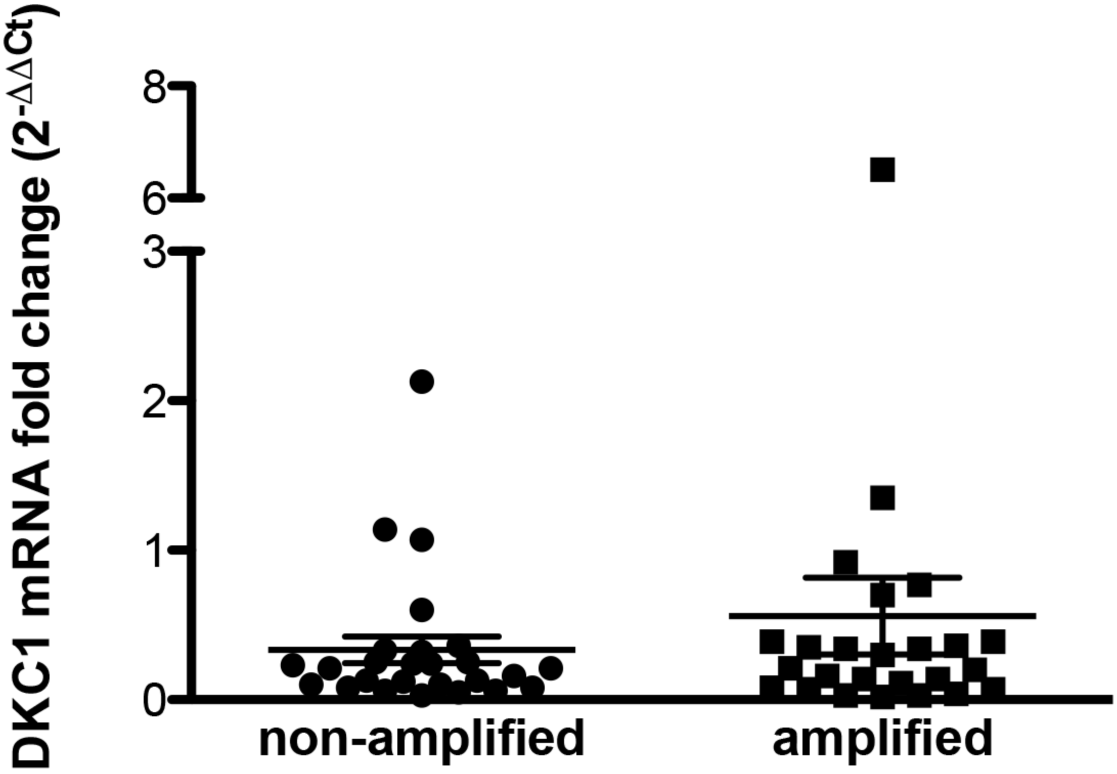

Supplementary Figure S1: DKC1 expression in *TERC* gene –amplified or –non-amplified lung cancers. Scatter plot graph showing DKC1 expression levels in the two series. DKC1 expression does not differ significantly in *TERC* amplified and non-amplified tumors, as determined by Student's *t* test (P = 0.387). DKC1 expression levels were measured by RT-PCR in cDNAs derived from two series of *TERC* gene –amplified and –non-amplified lung cancers, and compared to TERC expression in A549 cells.

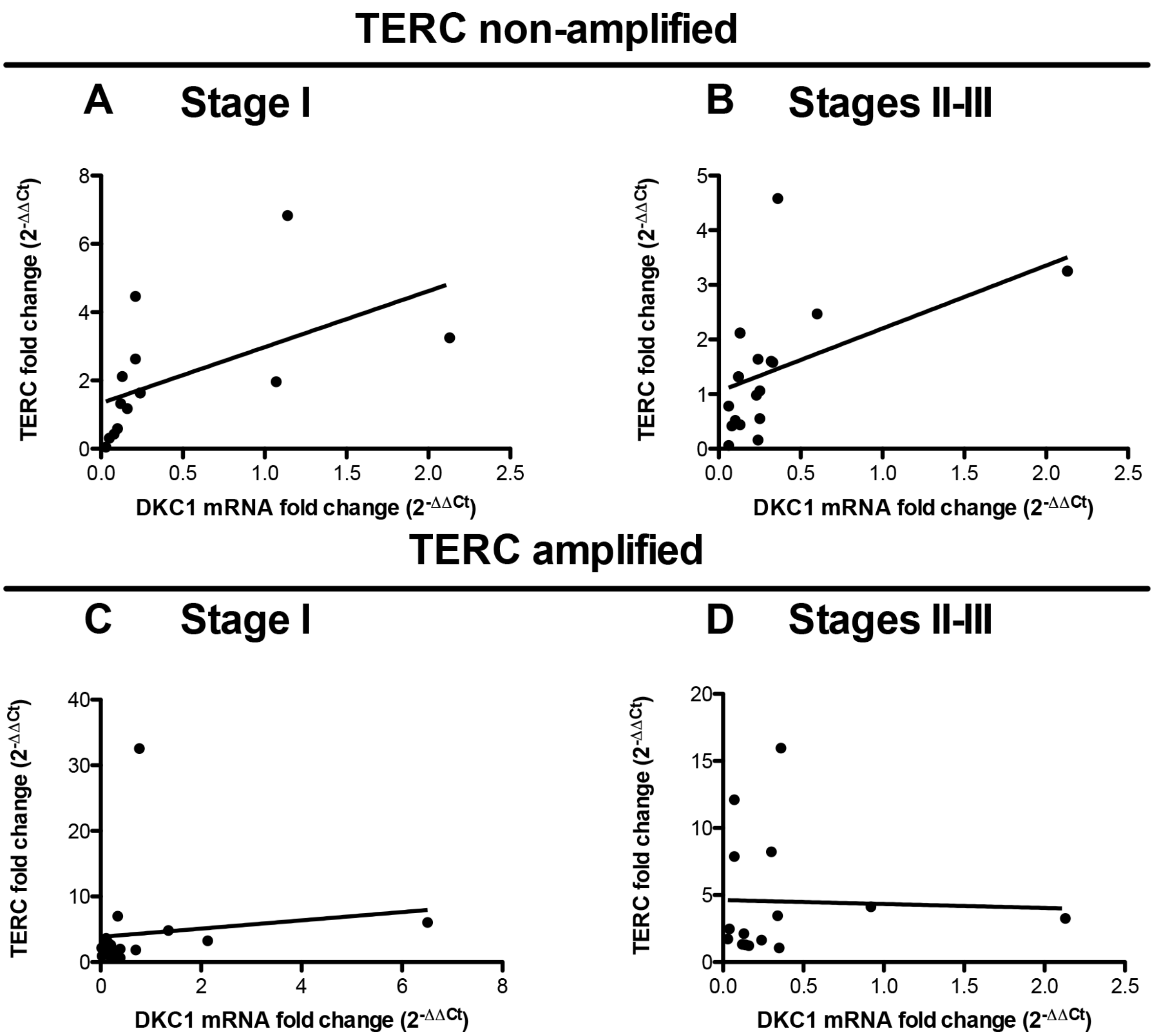

Supplementary Figure S2: TERC and DKC1 expression in TERC gene –amplified or –non-amplified lung cancers, divided by stage of the disease. A–D. Correlation, determined by linear regression analysis, between TERC and DKC1 expression is direct in those tumors where TERC gene is not amplified, and this correlation is maintained in stage I ($P = 0.045$) (A) and stages II–III ($P = 0.006$) (B) whereas there is no correlation in those tumors where TERC locus is amplified, independently of the stage ($P = 0.963$ and $P = 0.901$, respectively) (C. and D.) TERC and DKC1 expression levels were measured by RT-PCR in cDNAs derived from two series of *TERC* gene –amplified and –non-amplified lung cancers, and compared to TERC expression in A549 cells.
